# From Transmission to Transition: Lessons Learnt from the Thai Paediatric Antiretroviral Programme

**DOI:** 10.1371/journal.pone.0099061

**Published:** 2014-06-03

**Authors:** Olivia Tulloch, Sally Theobald, Jintanat Ananworanich, Sanchai Chasombat, Pope Kosalaraksa, Thidaporn Jirawattanapisal, Sudrak Lakonphon, Pagakrong Lumbiganon, Miriam Taegtmeyer

**Affiliations:** 1 Department of International Public Health, Liverpool School of Tropical Medicine, Liverpool, United Kingdom; 2 SEARCH and HIV-NAT, The Thai Red Cross AIDS Research Center, Bangkok, Thailand; 3 Faculty of Medicine, Chulalongkorn University, Bangkok, Thailand; 4 Bureau of AIDS, Tuberculosis and Sexually Transmitted Infections, Department of Disease Control, Ministry of Public Health, Nonthaburi, Thailand; 5 Department of Pediatrics, Faculty of Medicine, Khon Kaen University, Khon Kaen, Thailand; University of Cape Town, South Africa

## Abstract

**Background:**

The Thai HIV programme is a leader in the public health approach to HIV treatment. Starting at transmission of HIV and ending with transition to adult services this paper assesses the paediatric HIV treatment continuum from three perspectives: service-user, provider and policy maker, to understand what works well and why.

**Methods:**

A qualitative research design was used to assess and triangulate the stakeholder perspectives. Semi-structured interviews were conducted with ART service-users (n = 35), policy actors (n = 20); telephone interviews with prior caregivers of orphans (n = 10); and three focus group discussions with service-providers (hospital staff and volunteers) from a district, provincial and a university hospital.

**Findings:**

Children accessing HIV care were often orphaned, cared for by elderly relatives and experiencing multiple vulnerabilities. Services were divided into three stages, *1. Diagnosis and linkage*: Despite strong policies there were supply and demand-side gaps in the prevention of mother-to-child transmission ‘cascade’ preventing early diagnosis and/or treatment. *2. Maintenance on ART* - Children did well on treatment; caregivers took adherence seriously and valued the quality of services. Drug resistance, adherence and psychosocial issues were important concerns from all perspectives. *3. Adolescents and transition*: Adolescent service-users faced greater complexity in their physical and emotional lives for which providers lacked skills; transition from the security of paediatric clinic was a daunting prospect. Dedicated healthcare providers felt they struggled to deliver services that met service-users' diverse needs at all stages. Child- and adolescent-specific elements of HIV policy were considered low priority.

**Conclusions:**

Using the notion of the continuum of care a number of strengths and weaknesses were identified. Features of paediatric services need to evolve alongside the changing needs of service users. Peer-support volunteers have potential to add continuity and support at all stages. It is critical that adolescents receive targeted support, particularly during transition to adult services.

## Introduction

Thailand has a concentrated HIV epidemic with a seroprevalence of less than one per cent in antenatal sentinel surveillance sites and an estimated 14,000–35,000 infected children in 2011 [1], [2]. It was one of the first middle-income countries to introduce free, comprehensive HIV treatment for children [3]. Since 2000 antiretroviral therapy (ART) for the prevention of mother-to-child transmission (PMTCT) and routine testing in antenatal care (ANC) has been available [4], [5]. In this successful and evolving programme government data showed a sharp drop in MTCT, ranging between 3% and 6% from 2004–2008, depending on protocol compliance [6]. However, HIV positive women are less likely to attend ANC (87% vs 95%) [4], many children continue to present for diagnosis and treatment late [7] and treatment is only available for Thai nationals. As in other settings, optimal outcomes require full participation in the PMTCT care cascade [8], [9] and treatment from early childhood [10]. Thai children residing in orphanages have shown better clinical profiles at presentation and lower mortality than those living with parents or relatives [11]. Long-term viral suppression in children is influenced not only by clinical factors but also psychosocial issues [7], [12]–[14] including vulnerabilities to poverty and stigma [15], risk of a range of psychosocial problems is often heightened in adolescence [16].

In Thailand HIV services for children are delivered by the Ministry of Public Health through a holistic model, which incorporates clinical and psychosocial aspects. These services are often provided together at ‘one-stop clinics’. They include consultations with a nurse, paediatrician, pharmacist and social worker; clinical monitoring and volunteer-group activities. The volunteers are people living with HIV (PLHIV) organized through a national network. Hospitals may provide home visits, life-skills and HIV education and activities to raise community awareness. Children usually initiate ART at tertiary (provincial) hospitals and can be referred back to a district hospital when their health becomes stable [17]–[19]. Transition to adult clinic should normally occur at age 15.

The Thai paediatric ART programme is an innovative and holistic model not previously evaluated. We set out to investigate the experiences of services in the care continuum, from transmission of HIV through to transition to adult services. We sought the multiple perspectives of service-users, service-providers and ‘policy actors’ to better understand what works well and why and to learn lessons that can inform programme evolution.

## Methods

### Study design

A multi-method qualitative research design was used to assess and triangulate a range of perspectives on paediatric HIV services. Rigorous qualitative methods in HIV research are valued for bringing in-depth understanding to the patient experience, and recognition of the important influence of contextual factors that occur at intra- and interpersonal, community, social, cultural, and economic levels [20]. Data were collected in 2009. Service-provider and service-user participants were recruited from a university, a provincial and a district hospital in Khon Kaen Province, Northeast Thailand and two HIV orphanages in Lopburi province, Central Thailand. The orphanages were selected in a different province for reasons of convenience and availability of data, the orphans originated from all regions of the country. We conducted semi-structured interviews with ART service-users (n = 35), and policy actors (n = 20); telephone interviews with prior caregivers of orphans (n = 10); and three focus group discussions with service-providers (Table 1).

**Table 1:** Type and source of data collected.

Site	Service-user interviews	Policy actor interviews	Service provider FGD
**Type**	29 – caregivers	4 – NGOs	1 – District hospital (8 participants)
	6 – adolescents	2 – International agencies	1 – Provincial hospital (11 participants)
	10 – prior caregivers	5 – Academic/Expert	1 – University hospital (12 participants)
		9 – Government	
**Total**	**45**	**20**	**3**

### Service-user interviews

Paediatric ART clinic service-users (caregivers and older children) participated in qualitative interviews using a semi-structured guide designed to elicit detail. Information was asked about family and socio-economic situations, HIV support structures, stigma, HIV education, perceptions about services, challenges related to HIV and changes over time. The interviews were carried out in Thai or local Northeastern dialect by a female PLHIV researcher. Participants were selected purposively by the HIV care teams on clinic days to represent a range of different experiences including: adolescence; orphanhood; a range of income levels; adherence issues; experienced social exclusion, stigma or abuse; psychosocial difficulties or isolation; HIV disclosure issues. Registered patients who had not experienced any of these HIV related difficulties were rare; effort was made to ensure positive and negative experiences were elicited.

Interviews also took place with prior caregivers of orphans living at orphanages. Using convenience sampling participants were invited for telephone interview if contact information was still available to discuss perceptions about HIV and service availability. Interviews were undertaken in Thai by a member of orphanage staff with whom caregivers were acquainted.

### FGDs with service-providers

The FGDs, held with 8–12 participants, comprised hospital staff and volunteers at each hospital included in the study. All members of the paediatric HIV team were invited, including paediatricians, nurses, pharmacists, social workers and PLHIV volunteers from peer support groups. Areas explored included: guidelines, clinic procedures, provider and patient challenges in paediatric services and the national Children's ART Network. FGDs were used to understand how group norms and dynamics shaped experiences amongst the teams [21], [22], they were conducted in Thai by experienced Thai facilitators and the primary author.

### Interviews with policy actors

Policy actors were purposively sampled (n = 20) to capture the full range of perspectives and experience in the policy process, additional respondents were recruited through snowball sampling (Table 1). Interviews were conducted by the lead author in Thai and/or English.

### Analysis

All qualitative data were recorded, transcribed and translated to English then analysed using a thematic framework in QSR Nvivo (v8) software. Emerging themes were grouped and coded [23]. Trustworthiness and validity of the qualitative data were ensured through triangulation of the results between FGDs and interviews and between types of respondents. This enabled a multi-dimensional understanding to the issues [24] and resolution of contradictions [25]. Preliminary themes and analyses were presented to providers (staff and volunteers) in two workshops to check for validity and obtain feedback which was used to structure the final refined coding frame. The workshops also focused on identifying common gaps and future priorities for services.

### Ethics Statement

The research protocol received ethical approval by the Liverpool School of Tropical Medicine (Protocol No. 07.57), the Thai MOPH (Protocol No. 150/2551), Khon Kaen University (for Srinagarind Hospital) and Khon Kaen Provincial Hospital (also covering the district level hospital). Written consent was obtained directly from participants, including young participants (≥12 years). Young participants who attended clinic unaccompanied did not require additional consent from a caregiver, written consent was obtained from a caregiver for those who were accompanied. Consent was obtained verbally for participants in telephone interviews.

## Results

We first present a profile of service-users, followed by findings from three main stages of the paediatric ART continuum, (diagnosis and linkage to care; maintaining children on ART; and, adolescence and transition). Each stage incorporates data from all respondent types to show where views differ or confer and includes annotated illustrative quotations.

### Service-user profile and circumstances

The service-user and provider data created a profile of families largely living in poverty, vulnerability, isolation and low education level of caregivers, many of whom were elderly. Caregivers often appeared to struggle to care for children whose parents worked away or had died and one tertiary hospital worked with a large number of children living in orphanages. For example,

*“The children we work with often don't live with their parents, but elderly caregivers; their ability to build the child's potential is limited, the economic side of things is often tough which reduces the ability to give the child the opportunities they need.”* (FGD, Provider)

*“We are labourers, we don't have any land, whatever [work] is available we will go to.”* (Interview, Father)

*“The doctor explains things to the child too…I'm forgetful… and I'm getting old.”* (Interview, Grandmother)

Caregivers' accounts revealed that many had positive and pragmatic approaches to doing their best for the HIV-positive child. Attitudes of acceptance or resignation were spread across the sample. They generally took their responsibilities seriously and were committed to regular clinic attendance.

*“I don't think she'll live that long…all I think about is finding enough money so that she can have some happiness.”* (Interview, Mother)

*“Even if I'm dying, or unwell I know I have to bring her to the appointment!”* (Interview, Grandmother)

The telephone interviews with prior caregivers described a shift in service-user experience and decision-making about care for children with HIV since ART became freely available. Previously stigma, chronic ill-health and lack of treatment options were major reasons for relatives sending children to orphanages. Later, drug resistance, difficulties with treatment adherence and coping with adolescent behaviour had become more likely reasons. For example:

*Child sent pre-free ART: “I didn't think she would survive, but I sent her [to the orphanage].”* (Telephone interview, Grandmother)

*Children sent post-free ART: “It was fine when his mother was here to make him take it [ART] on time…but afterwards, sometimes he'd take it on time, sometimes he wouldn't. I'd even have to hit him to make him take it.”* (Telephone interview, Father)

*“We thought that we would try to continue caring for him ourselves… but it was too difficult because of the discrimination at school.”* (Telephone interview, Grandmother)

### Stage One. Diagnosis and linkage to care

There was evidence from multiple perspectives of early infant diagnosis (EID) and linkage to treatment services not occurring. Some mothers had avoided ANC and this was coupled by avoidance or failure to access HIV follow-up services during infancy. Demand-side reasons that were identified for failure to access timely prevention or treatment services included parents working away from home, denial of HIV status and feelings of hopelessness: Representative examples include:

*“I was working in the South, I didn't have a health card for the hospital there. … I wanted to die, I didn't want to exist.”* (Interview, Mother)

*“His mother knew she was infected so she didn't go to hospital to give birth.”* (Interview, Grandmother)

HIV positive children usually did not access services until much later and only three interviewees cared for children who had remained in the system from infancy until treatment initiation. There was anecdotal evidence of children, including some without official Thai nationality, who had not accessed services; and several service-users knew of untreated HIV-positive children.

*“Last week we had a newly diagnosed 11 year old girl. The father kind of knew but ignored it… I believe there a lot [of HIV positive children] who have not come in to the system.”* (FGD, Provider)

*“I know someone in the village whose grandmother won't bring her to be treated. The child is not well… I don't know why she won't. I've told her all about the treatment.”* (Interview, Father)

In contrast to service-users and providers, policy actors tended to be have a high opinion about existing PMTCT and linkage to care, partly due to published projections of reduced perinatal HIV transmissions (described in the Asian Epidemic Model [26]).

*“You must think that it is a temporary phenomenon, the paediatric HIV.”* (Interview, Policy actor)

These respondents believed that most HIV-positive children were receiving ART according to national guidelines. Some MTCT experts were concerned however, and felt that HIV positive women and non-Thai migrants were less likely to receive ANC and so miss the opportunity to link to HIV services.

*“The [HIV positive] ANC group has about 0.8% infection rate, whereas those who don't have ANC have between 3% and – in some areas – 5%… infection rate, we need improvements.”* (Interview, Policy actor)

### Stage two. Maintaining children on ART

Respondents were asked about their experiences of paediatric ART services. Service-users' perceptions of clinic services and volunteer support were generally good and they felt they received both knowledge and reassurance.

*“The staff are proud to work here, they supervise the care well.”* (Interview, Grandmother)

*“There is one volunteer I have known since the start, she has given me helpful information, she said she is also infected and … she helped me to understand.”* (Interview, Aunt)

Many caregivers however, had concerns about caring for children living HIV, such as the recurrent health problems, disclosure, adherence or drug resistance.

*“This is the last chance for her; there aren't any other drugs available.”* (Interview, Father)

Service-providers described how caregivers and patients often faced multiple non-clinical challenges that influenced their capacity to follow the HIV treatment-and-care continuum. They were concerned about the impact of family instability, poverty and poor education and attitudes.

*“You need to be able to separate the issues, I'd give it two sides, the physical – dealing with illness and hospitals – and the psychosocial side, which can involve lack of love. We're dealing with poor people who don't care much about the psychological factors; they prioritize the physical aspects. But everyone needs love, especially these children.”* (FGD, Provider)

*“It is most important for them [caregivers] to understand not just that they have AIDS, but how they can look after themselves, what processes they need to prepare for. These issues, the care and disclosure issues need to be absorbed through activities, it isn't enough just to speak to them about it.”* (FGD, Provider)

Case-conferencing, home visits and social assessments were considered optimal but not all sites felt able to implement these. Most staff – particularly at district and provincial level – felt they lacked capacity and tools to assess service-users needs accurately.

*“We feel we aren't that good. Well, we need to receive more training and have a clearer system… and to improve our confidence for things like counselling.”* (FGD, Provider)

Clinic staff and volunteers expressed strong commitment to their work and patients. They often felt overburdened and some thought that HIV clinics would benefit from clearer coordination. Successful one-stop clinic organization was deemed to depend on cohesion, efficiency and communication between team members to deal with patients' multiple needs.

*“We need to assess what the problems are in our clinic and see what the issues are … Then we can form a plan - like a system that starts when they arrive at the clinic the first time, but follows them home to the issues there too. It would be good to have information about each child, including a map of where their house is, family issues, communication to date; so that if the child stops coming or the caregiver doesn't come to get the meds we can follow them.”* (FGD, Provider)

The volunteer peer support groups were perceived by formal hospital staff to have an essential role in enhancing the quality of the service. Volunteers themselves appeared extremely motivated; asked why she volunteered one team member responded “*but how could we* ***not*** *do it?!*” (FGD, Provider)

Policy actors from the government sector generally felt that the ART programme was a successful, progressive model. Lack of paediatric specific policies, monitoring and evaluation were identified as important weak points that would inhibit future developments. Policy actors were not necessarily aware of the lack of capacity or challenges described by providers, although current policy recommends technical support of district level provision of HIV care from the tertiary level,

*“With this [training], telephone and email communication the district hospitals can manage… Why can't you have a manual with back-up consultation? Nurses can do it, usually the Thai public health system is managed by nurses.”* (Interview, Policy actor)

### Stage three. Adolescence and Transition

The adolescents interviewed (Table 1) responded positively about their paediatric clinic experiences, could talk knowledgably about HIV and valued clinic staff (including volunteers). Other older children however, were apparently confused or suffering from the lack of clarity or disclosure about their condition, as described by this caregiver:

*“He says ‘My CD4 is only 2%', so he knows what it is, but he doesn't know that it refers to HIV… Or, maybe he does know, maybe that is why he gets so angry”* (Interview, Aunt)

Adolescent care was a clear concern for service-providers due to the complex requirements of young people with HIV. They observed growing proportions of teenagers with drug resistance and felt that the many elderly caregivers lacked the capacity to deal with adolescent behaviour.

*“All teenagers have some degree of problems, but these ones also have HIV, and so their problems are intensified.”* (FGD, Provider)

Attendance at HIV ‘life-skills’ camps, organized by clinic teams, were considered as a important source of information, moral support and enjoyment for older children who knew their HIV status. Despite this some providers still felt inadequately equipped to support them, describing a lack of training or capacity with which to teach adolescents about sex, relationships and responsibility; difficulties dealing with disclosure to adolescents who had had their status hidden from them; unreliable adherence due to boredom with ART or behavioural problems; and psychosocial problems resulting from neglect, abandonment or HIV status.

*“We have hardly any tools for this [communication with patients] at all, really very few… We don't see anything new like for example, how to deal with teenagers”* (FGD, Provider)

Those policy actors with insight into adolescent HIV issues, (in particular NGO respondents) were vocal about the lack of special provision of targeted services for adolescents,

*“We hear frequently from organisations who are working with HIV-positive kids that then become adolescents, [they say] that they can't do anything for them anymore”* (Interview, Policy actor)

All participant groups recognized the difficulty of transition from paediatric to adult clinic. Service-users and providers concurred that adolescents were comfortable at the paediatric clinic. The good and/or long-established rapport with the teams, meant they were unprepared to leave its protective comfort at the age of 15 years:

*“Oh no, I want it to be like this. The doctor suggested it [transition] before, but if I went there [adult clinic], I wouldn't be able to meet with my friends or all the other aunties or the same doctor” (Interview, adolescent aged 17)*

PLHIV volunteers and staff who straddled both clinics were suggested as potential ways to support adolescents by providing continuity through the transition process.

## Discussion

The relationships between paediatric HIV services, service-users' experiences of HIV and their care-seeking behaviour are complex and not well understood in Asia. Using a qualitative methodology we have described the stages of the journey through paediatric HIV infection from different perspectives. Our assessment identifies the innovative elements of the holistic Thai system and enables identification of weak and strong aspects of service delivery. It is a progressive, patient-centred and holistic approach to paediatric HIV services that incorporates PLHIV support to link clinics and communities. The current patient profile is generally one of children diagnosed with HIV late, whose needs and family circumstances make them more vulnerable than their peers in the general population. The data suggest that experiences faced today are different from when free ART was first available due to shifts in expectations, community attitudes, and the needs of a cohort which is increasingly in adolescence. Key challenges are gaps in PMTCT coverage and linkage to treatment services, difficulties balancing psychosocial needs with clinical needs, and limited strategies to support adolescents into adulthood.

Service-users in this study had high opinions of the care they received and displayed commitment to regular follow-up. However there was evidence of lost opportunities the ante- and postnatal care cascade (the multiple steps in ante- and post-natal services) and some mothers in vulnerable groups failed to receive PMTCT or linkage to treatment services. Well-functioning interventions for PMTCT can almost eliminate new infant infections [27], but loss to follow up-from the care cascade is high in many settings [8]. In Thailand PMTCT protocols are in line with the most recent WHO recommendations. A recent Thai study however, shows compliance with protocols is low and MTCT rates were higher among partial or non-compliant groups than compliant groups (transmission to 6%, 9.5% and 1.2% respectively). Compliance was lower among non-Thais and those without prior knowledge of HIV status [6]. Limited data from other studies show higher HIV prevalence in non-Thais (1–9%), and lower ANC attendance in this group [28] and HIV-infected women [28], [29]. Our findings are consistent with these studies and suggest that specific targeting of services towards vulnerable and mobile populations [6] is still needed alongside stronger links between antenatal and postnatal services to prevent continued loss to follow-up from the care cascade.

Clinic staff often struggled to deliver a service that met the complex needs of children on ART; PLHIV volunteers who act as ‘co-providers' at clinic and in the community made a substantial contribution and were highly valued. In Thailand they are a recognized part of the strategy to ensure long-term psychosocial support in HIV care [19], [30]. Our assessment highlights the need for providers to develop broader skills to cope with the ever-changing physical and psychosocial burden of HIV infection in a vulnerable population with relatively weak support structures. In addition to HIV clinical management, they need to be responsive over time to the changing personal circumstances faced by patients outside clinic. Improved paediatric-specific counselling tools, training and coordination were suggested to improve quality of services, this would ensure accurate child assessment and strengthen links between the clinic and home life. Expanding availability and training for existing Thai specific tools such as paediatric HIVQUAL-T [31], the paediatric disclosure model [32] and the quality of life assessment [33] could improve service-providers' capacity to consistently give care of good quality. Poor HIV health outcomes have been linked to poor quality of life in Thai children [33], and data show that supporting caregivers improves emotional intelligence of HIV-infected children [34]. Our study shows that volunteers can be seen a vital part of this support, they complete the holistic approach and would be consistent with WHO recommendations to shift tasks to less specialised workers [35].

In this study adolescents were shown to have complex support needs and transition to adult clinic was not occurring when it should. The Thai context is strengthened by the existence of volunteers assisting with multiple aspects of care but their potential role during transition is not fully exploited. As in other settings they may be involved in the stages of a phased approach to transition; protocols for this may include a timeline, checklists, an intermediary ‘teen clinic’ and life-skills activities [36], [37]. In a phased approach individuals can be gradually introduced to the prospect of transition and later accompanied to adult clinic until they are accustomed to it. Preparation should start early and be multidisciplinary so as to incorporate individuals' cognitive development and mental health, medication adherence, sexual and reproductive health, socioeconomic issues, stigma and disclosure [38].

The Thai paediatric HIV programme is a highly evolved model, in which providers have longer experience of providing ART to children than in most other resource-limited settings. There is a need for contextual understanding and a holistic approaches to HIV care that are grounded in service providers' and service users' experiences, and the local challenges and priorities [39]. The perspectives of participants in this study illustrated where gaps in the continuity of a paediatric HIV treatment and care continuum are likely to arise and the need to continually reassess the needs of HIV positive children in a changing epidemic. We found that the concept of a holistic approach was supported by service-providers and appreciated by service-users, but needed clearer policy backing. In 2013 69% of paediatric patients registered at the three sites at the time of this study would have reached (or passed) the age at which transition to adult services should occur; there is an urgent need to make provision for these cases. A well-coordinated team comprising hospital staff and volunteers can help to overcome human resources constraints and provide the continuity that children and adolescents will need as a new generation of HIV positive adults. The need for additional guidance for the care of adolescents is now being recognized in Thailand and some recommendations have been incorporated into ART guidelines [17]. Child- and adolescent-specific elements of HIV policy were considered a low priority and may contribute to the gaps in service provision that were visible at both ends of the care continuum in this study.

Our study has several limitations. Data was collected at a single point in time and therefore only provides a superficial sense of important changes over time. Respondents only included only those who are enrolled in care, the perspectives of those who have poorer access to services or avoid HIV services are important if the system is to be responsive to their needs. Service-user respondents were selected by health care workers, interviewed at health facilities and were recruited from a cultural group previously documented as averse to expressing criticism [40], [41]; efforts were made to mitigate bias but it may be that respondents were unwilling to criticise. It was possible to recruit few adolescents for interview, therefore the service-user perspective is largely one of adults used as proxy respondents for children.

Thailand was the first country in the region to provide free treatment to all clinically eligible children at the point of service. Using the notion of a continuum of care a number of strengths and weaknesses can be identified. Features of paediatric services need to be responsive to the evolving needs of service users. Peer-support volunteers have potential to add continuity and support at all stages. There is a need to address missed opportunities for early paediatric treatment, and it is critical that adolescents receive targeted support, particularly during transit to adult services. Other settings may learn from the strengths *and* weaknesses of the Thai system to deal with features which are increasingly common in other lower- and middle-income countries as the epidemic continues to evolve.
